# Insulin-like growth factor 1 is not associated with high myopia in a large Japanese cohort

**Published:** 2013-05-21

**Authors:** Masahiro Miyake, Kenji Yamashiro, Hideo Nakanishi, Isao Nakata, Yumiko Akagi-Kurashige, Akitaka Tsujikawa, Muka Moriyama, Kyoko Ohno-Matsui, Manabu Mochizuki, Ryo Yamada, Fumihiko Matsuda, Nagahisa Yoshimura

**Affiliations:** 1 Department of Ophthalmology and Visual Sciences, Tokyo, Japan; 2 Center for Genomic Medicine, Kyoto University Graduate School of Medicine, Kyoto, Tokyo, Japan; 3 Department of Ophthalmology and Visual Science, Tokyo Medical and Dental University, Tokyo, Japan

## Abstract

**Purpose:**

To investigate whether genetic variations in the insulin-like growth factor 1 (*IGF-1*) gene are associated with high myopia in Japanese.

**Methods:**

A total of 1,339 unrelated Japanese patients with high myopia (axial length ≥26 mm in both eyes) and two independent control groups were evaluated (334 cataract patients without high myopia and 1,194 healthy Japanese individuals). The mean axial length (mm±SD) in the case group was 29.18±1.85 mm, and the mean spherical equivalent (D±SD) of the phakic eyes was −12.69±4.54 D. We genotyped five tagging single nucleotide polymorphisms (SNPs) in *IGF-1*: rs6214, rs978458, rs5742632, rs12423791, and rs2162679. Chi-square tests for trend, multivariable logistic regression, and haplotype regression analysis were conducted.

**Results:**

We found no significant association between the *IGF-1* SNPs and high or extreme myopia (axial length ≥28 mm in both eyes, 837 subjects) in the additive model, even when compared with the cataract and general population controls, with or without adjustments for age and sex. The evaluation using dominant and recessive models also did not reveal any significant associations. The haplotype analysis with a variable-sized sliding-window strategy also showed a lack of association of *IGF-1* SNPs with high or extreme myopia.

**Conclusions:**

The results of the present study using a Japanese subset do not support the proposal that the *IGF-1* gene determines susceptibility to high or extreme myopia in Caucasians and Chinese. Further studies are needed to confirm our reports in other populations and to identify the underlying genetic determinants of these ocular pathological conditions.

## Introduction

Myopia is a common visual disorder found worldwide and poses major public health concerns, especially in East Asian populations. Myopic eyes with long axial lengths (≥26 mm) or a high degree of myopic refractive error (≤−6 D) are classified as high myopia [1]. High myopia is associated with various ocular complications [2], and these pathological conditions are one of the leading causes of legal blindness in developed countries [3-5]. Therefore, elucidating the pathological mechanisms underlying high myopia and discovering methods for preventing or delaying its onset are important.

Myopia is a complex disease caused by environmental and genetic factors. To date, although many studies have evaluated various candidate genes and susceptible loci of high myopia [6-11], no single gene has been consistently responsible for the condition. In addition to candidate gene studies, a genome-wide approach has also been performed by several groups. Our group previously determined a susceptibility locus for pathological myopia in 2009, using a genome-wide association study [12]. In addition, recent genome-wide association studies have revealed myopia susceptibility loci on chromosome 15 [13,14], and we confirmed that these susceptibility loci are also present in high myopia [15]. However, susceptibility genes for myopia have not yet been determined.

Insulin-like growth factor 1 (*IGF-1*) is similar to insulin in function and structure and is a member of a protein family involved in mediating growth and development. Recently, a single nucleotide polymorphism (SNP) in *IGF-1* was reported to be associated with several types of myopia, including high myopia, in Caucasians [16]. However, these associations were not confirmed by a Polish family study that used single-marker association analysis, a family-based association test, a pedigree disequilibrium test, and haplotype analysis [17]. Nevertheless, subsequent Chinese studies reported a significant association of *IGF-1* polymorphisms with high or extreme myopia; Mak et al. [18] reported an association with high myopia according to haplotype analysis but not single-marker analysis, and Zhuang et al. [19] reported an association with extreme myopia but not with high myopia according to the single-marker and haplotype analyses. The *IGF-1* gene is located at a well replicated myopia susceptibility locus, MYP3 [20-23]. Since several previous animal studies indicated that insulin and *IGF-1* were involved in myopia development [24-26], resolving these conflicting results and clarifying whether *IGF-1* polymorphisms are indeed associated with high myopia are essential. In the present study, we conducted a systematic case-control study to validate the association between polymorphisms of the *IGF-1* gene and high and extreme myopia, using a large cohort of 2,867 unrelated Japanese individuals.

## Methods

### Subjects

A total of 1,339 unrelated Japanese patients with high myopia who had agreed to participate in genomic study were recruited from the Kyoto University Hospital, Tokyo Medical and Dental University Hospital, Fukushima Medical University Hospital, Kobe City Medical Center General Hospital, and Ozaki Eye Hospital. All the patients underwent a comprehensive ophthalmic examination, including dilated indirect and contact lens slit-lamp biomicroscopy, automatic objective refraction, and measurements of the axial length by applanation A-scan ultrasonography or partial coherence interferometry (IOLMaster, Carl Zeiss Meditec, Dublin, CA). To be classified as having high myopia, the subjects had to have an axial length ≥26 mm in both eyes. Of the 1,339 patients with high myopia, 837 had extreme myopia, which is defined as an axial length ≥28 mm in both eyes.

As control subjects, two cohorts were included. One cohort was composed of selected controls, comprising 334 cataract patients with axial lengths <25.0 mm in both eyes (control 1). These patients were recruited from the Department of Ophthalmology at Kyoto University Hospital, Ozaki Eye Hospital, Japanese Red Cross Otsu Hospital, and Nagahama City Hospital. The axial length was measured with applanation A-scan ultrasonography or partial coherence interferometry before cataract surgery, and dilated fundus examination was performed after surgery. If the fundus examination results revealed myopic changes such as lacquer cracks/peripapillary atrophy, staphyloma, or choroidal neovascularization, the subject was eliminated from control 1. The other cohort was composed of general population controls, comprising 1,194 healthy Japanese individuals recruited from the Aichi Cancer Center Research Institute, who had agreed to participate in genomic study (control 2).

All the procedures adhered to the tenets of the Declaration of Helsinki. The institutional review board and ethics committee of each participating institute approved the protocols. All the patients were fully informed of the purpose and procedures of the study, and written consent was obtained from each patient.

### DNA extraction

Total genomic DNAs were prepared from 14 ml of venous blood. DNA was purified using a DNA extraction kit (QuickGene-610L, Fujifilm, Minato, Tokyo, Japan).

### Single nucleotide polymorphism selection and genotyping

We selected tag SNPs in *IGF-1* based on HapMap Phase II (Build 36) genotype data [27] using the Haploview software (ver. 4.2). Although previous studies tagged relatively minor SNPs (minor allele frequencies [MAFs] ≥0.05 or 0.10 were applied), all such minor SNPs showed no association with high or extreme myopia. In addition, rs6214 and rs12413791, which were reported to be associated with high and extreme myopia, respectively, showed a MAF of 43% and 31% in HapMap JPT (Japanese in Tokyo, Japan). Thus, we tagged all the major (MAF ≥30%) SNPs that showed a Hardy–Weinberg p≥0.05. Using a tagger pairwise program provided in Hapmap project (R2 cutoff of 0.90), we selected five SNPs: rs6214, rs978458, rs5742632, rs12423791, and rs2162679. These tags provided 100% coverage of the major HapMap SNPs within an 84.65-kilobase region spanning the *IGF-1* gene.

The samples of the high myopia cases and cataract controls were genotyped using a commercially available assay (TaqMan SNP assay with the ABI PRISM 7700 system; Applied Biosystems, Foster City, CA). The individuals recruited from the Aichi Cancer Center Research Institute were genotyped using Illumina HumanHap 610 chips (Illumina Inc., San Diego, CA). Because rs12413791 was not included in this chip, the genotype for rs12423791 was imputed using the MACH software, based on the HapMap Phase II JPT genotype data.

### Statistical analyses

Deviations in genotype distributions from the Hardy–Weinberg equilibrium (HWE) were assessed for each group with the chi-square test. The chi-square test for trend or its exact counterpart was used to compare the genotype distributions of the two groups. To adjust for age and sex, we performed a multivariable logistic regression analysis. In addition, dominant and recessive models were also calculated using the chi-square test. These statistical analyses and power calculation were performed with R software (R Foundation R 2.13.0 for Statistical Computing, Vienna, Austria) and PLINK software (ver. 1.07). We also conducted a haplotype analysis by using a variable-sized sliding-window strategy [28] using the PLINK software. p≤0.05 was considered statistically significant. The Bonferroni correction was used for multiple comparisons.

## Results

### Demographics of the study population

The demographic characteristics of the study population are shown in Table 1. The axial length of the 2,678 eyes of the 1,339 highly myopic cases ranged from 26.00 to 39.73 mm, with a mean ± standard deviation (SD) of 29.18±1.85 mm. Among these 2,678 eyes, 1,881 (70.2%) were phakic. Their mean refraction was −12.69±4.54 D. The axial length of the 668 eyes of the control 1 group ranged from 18.67 to 24.92 mm, with a mean±SD of 22.96±0.87 mm. The mean refraction of the phakic eyes in control 1 was −0.379±3.01 D. The high myopic cases were significantly older and more female-dominant than both control groups (p<0.001).

**Table 1:** Characteristics of the Study Population

Parameters	Patients		Control 1		Control 2	
	High myopia*	Extreme myopia*	Cataract†	P value	Population-based controls	P value
Patients, n	1339	837	334		1194	
Age in years, (mean±SD)	57.2±14.9	57.4±14.1	74.8±8.12	<0.001‡	50.3±15.9	<0.001‡
**Sex, n (%)**						
Male	442 (33.0%)	296 (35.1%)	132 (39.5%)	<0.001§	493 (41.3%)	<0.001§
Female	897 (67.0%)	547 (64.9%)	202 (60.5%)		701 (58.7%)	
**Axial length, (mm±SD)**						
Right eyes	29.25±1.87	30.18±1.56	22.94±0.88		NA	
Left eyes	29.12±1.83	30.06±1.49	22.97±0.85		NA	
**Refraction of the phakic eyes, D**						
Right eyes	−12.83±4.48	−14.41±4.41	−0.459±3.24		NA	
Left eyes	−12.55±4.60	−14.24±4.54	−0.294±2.76		NA	

* High myopia: axial length ≥26.00 mm in both eyes. Extreme myopia: axial length ≥28.00 mm in both eyes. † Individuals who underwent cataract surgery and who had an axial length <25.00 mm in both eyes. ‡ Unpaired *t* test. Compared with the high myopia group. §Chi-square test. Compared with the high myopia group.

### Genetic distribution

The genotype counts and HWE p value for the five SNPs in the high myopia, extreme myopia, and control groups are shown in Table 2. Because we calculated the HWE p values for 20 genotype distributions, the HWE p value cutoff was set to 0.0025, using the Bonferroni correction. Thus, the distributions of the genotypes for the five SNPs were all in HWE.

**Table 2:** Genotype Counts and Hardy–Weinberg Equilibrium P Value in the High and Extreme Myopia Cases and Controls

SNP name	Allele definition		High Myopia					Control 1				
	Allele 1	Allele 2	1/1*	1/2*	2/2*	Allele1 frequency	HWE p value	1/1*	1/2*	2/2*	Allele1 frequency	HWE p value
rs6214	C	T	277	641	373	0.463	0.955	83	159	88	0.492	0.51
rs978458	T	C	256	661	361	0.459	0.144	68	154	110	0.437	0.316
rs5742632	G	A	209	657	410	0.421	0.051	58	151	120	0.406	0.423
rs12423791	C	G	97	452	672	0.265	0.091	23	109	194	0.238	0.169
rs2162679	C	T	178	540	569	0.348	0.007	40	146	145	0.341	0.715
**SNP name**	**Allele definition**		**Extreme myopia**					**Control 2**				
	**Allele 1**	**Allele 2**	1/1*	1/2*	2/2*	**Allele1 frequency**	**HWE p value**	1/1*	1/2*	2/2*	**Allele1 frequency**	**HWE p value**
rs6214	C	T	179	392	223	0.472	0.776	268	585	341	0.469	0.562
rs978458	T	C	158	401	228	0.456	0.473	264	596	334	0.471	1
rs5742632	G	A	123	410	256	0.416	0.057	229	586	379	0.437	0.953
rs12423791	C	G	54	268	421	0.253	0.208	85	468	641	0.267	1
rs2162679	C	T	108	331	352	0.346	0.034	149	541	504	0.849	0.351

* 1/1: genotype with homozygous allele 1; 1/2: genotype with heterozygous alleles; 2/2: genotype with homozygous allele 2.

### Genetic association test

We evaluated the association between each SNP and high myopia using three models: additive, dominant, and recessive. The p values are presented in Table 3. Although no SNP showed a significant association with high or extreme myopia in any models when compared with control 1, the SNP rs5742632 showed a p<0.05 in the association with extreme myopia when compared with control 2 in the recessive model. However, this SNP did not show any associations after the multiple comparison correction. The other SNPs also showed no association with high and extreme myopia, in any of the models. The lack of association persisted even after an adjustment for age and sex. The adjusted odds ratios are shown in Appendix 1.

**Table 3:** Genetic Association Test for 5 SNPs (versus High Myopia/Extreme Myopia)

SNP name	Additive model	Additive model	Dominant model	Recessive Model
	Nominal p value*	Adjusted p value†	Nominal p value‡	Nominal p value‡
**Control 1**				
rs6214	0.175/0.389	0.294/0.456	0.424/0.637	0.150/0.341
rs978458	0.302/0.413	0.586/0.569	0.081/0.162	0.855/0.867
rs5742632	0.466/0.654	0.674/0.822	0.135/0.189	0.587/0.394
rs12423791	0.177/0.453	0.213/0.315	0.148/0.373	0.594/0.906
rs2162679	0.754/0.840	0.568/0.429	0.895/0.845	0.407/0.483
**Control 2**				
rs6214	0.642/0.857	0.745/0.889	0.855/0.805	0.552/0.971
rs978458	0.402/0.348	0.500/0.362	0.880/0.642	0.205/0.273
rs5742632	0.251/0.177	0.311/0.192	0.836/0.757	0.069/0.039
rs12423791	0.838/0.346	0.805/0.335	0.505/0.212	0.442/0.908
rs2162679	0.815/0.732	0.603/0.572	0.315/0.325	0.320/0.452

* Trend χ^2^ test. † Generalized linear model. Adjusted by age and sex. ‡ Generalized linear model.

We also conducted a haplotype analysis by using a variable-sized sliding-window strategy. This analysis also did not show any significant association. The lowest p=0.147, which was observed with a five-SNP window (Table 4). These haplotypes include the rs12423791-rs5742632 haplotype, which has been reported to be associated with extreme myopia.

**Table 4:** Variable-Sized Sliding-Window Haplotype Analysis.

Haplotypes	versus high myopia	versus extreme myopia
	lowest p value	lowest p value
**2-SNP window**		
rs6214-rs978458	0.425	0.497
rs978458-rs5742632	0.572	0.422
rs5742632-rs12423791	0.712	0.447
rs12423179-rs2162679	0.149	0.493
**3-SNP window**		
rs6214-rs5724632	0.246	0.53
rs978458-rs12423791	0.564	0.336
rs5742632-rs2162679	0.252	0.327
**4-SNP window**		
rs6214-rs12423791	0.298	0.306
rs978458-rs2162679	0.293	0.343
**5-SNP window**		
rs6214-rs2162679	0.147	0.373

The statistical power to detect an association of a risk allele with an odds ratio of 1.30 at a significance level of 0.01 is more than 99%. In addition, the statistical power calculation revealed that our sample size detected the gene-disease association for an odds ratio of 1.19 by more than 80%.

## Discussion

Here, we report a case-control study of the association of high and extreme myopia with several polymorphisms, using two Japanese control cohorts. All of the five tagging SNPs of *IGF-1*, including rs6214 and rs12423791, which were suggested to be associated with high or extreme myopia in previous studies, showed no association with high and extreme myopia in the additive, dominant, or recessive model.

*IGF-1* was identified as a high myopia-related protein in two animal model studies. These studies showed that IGF-1 or insulin injection into chick eyes accelerated their axial elongation, whereas glucagon injection decelerated it [24,25]. Penha et al. [26] indirectly supported this in 2011; they reported that hyperopic defocus, a cause of axial elongation of chick eyes, is associated with overexpression of the IGF-1 receptor in chick eyes. In humans, although a significant association of the *IGF-1* SNP with any myopia and with high myopia was reported in Caucasians in 2010 [16], the association was not confirmed in a Polish family cohort, using the single-SNP association, family-based association, and pedigree disequilibrium tests [17]. Furthermore, two subsequent Chinese studies showed a significant association of *IGF-1* tag SNPs with high or extreme myopia [18,19]. However, these replication studies have limitations due to the evaluation of a small cohort in each study (127, 300, and 302 cases, respectively). Small cohorts do not faithfully represent the larger population, which can lead to the generation of false-positive results. In addition, an analysis of a small cohort only has low statistical power, which can also give rise to false-negative results.

We conducted a systematic case-control study to evaluate the association of *IGF-1* with high myopia in the Japanese population, using a relatively large cohort of 1,338 high myopic cases, 334 cataract controls, and 1,194 healthy Japanese controls; the statistical power of the single-SNP analysis was quite high (≥99%). Despite the high statistical power, all of the five tagging SNPs selected to cover 100% of the major *IGF-1* SNPs showed no association with high and extreme myopia, though we fit all the hereditary models. Furthermore, haplotype analysis with the powerful variable-sized sliding-window strategy did not reveal any significant association with high and extreme myopia. The inclusion criteria for the present study are more suitable than those of the previous studies; we used axial length as an indicator of myopia, whereas the previous studies (except Zhuang’s study [19]) used spherical equivalent. In the chick study, insulin or IGF-1 injection into chick eyes accelerated their axial elongation [24,25], implicating both as possible causes of axial myopia, rather than refractive myopia. Given that the previous studies included refractive myopia cases, of which only a proportion are axial myopia, we infer that they lack the power to rigorously test the association between *IGF-1* SNPs and axial myopia.

At first glance, this result seems to conflict with the previously mentioned chick study [24,25,29]. However, this may be due to differences between avians and mammals. First, chick eyes have cartilage in the sclera, whereas mammalian eyes do not [30]. This structural difference may affect the response to IGF-1 because this hormone is also proposed to affect the anabolism of cartilage matrix molecules [31]. Second, the biochemistry and signaling cascades from the retina to the sclera differ between chicks and mammals. For instance, glucagon, which plays an important role in inhibiting myopia in the chick model, had no effect in a mouse model [29,32]. Similarly, the all-trans-retinoic acid levels in the retinal pigment epithelium during induction of myopia were inversely proportional in chick and marmosets [33,34]. Therefore, it is possible that IGF-1 has an effect on the development of myopia in birds but not in humans.

Our study does have some limitations. The first is the SNP selection. Although the five evaluated SNPs covered 100% of all the major *IGF-1* SNPs, we targeted only major SNPs. Relatively minor and untaggable SNPs might be functionally important in the onset or pathological mechanism of high myopia. However, although the original study [16] evaluated a total of 13 tagging SNPs that showed a MAF ≥5% in the *IGF-1* gene, the study reported that only rs6214, whose MAF is 42%, showed a significant association with high myopia. Furthermore, the subsequent Chinese studies evaluated tagging SNPs that showed MAFs ≥10% in the *IGF-1* gene, and only rs12423795, whose MAF is 31%, showed a significant association [18,19]. Hence, it seems futile to evaluate minor SNPs when testing the association of *IGF-1* SNPs with high myopia. Second, we did not trace the method of Mak’s study, which showed a significant association of *IGF-1* haplotypes with refractive high myopia. Accordingly, we cannot negate the association, in the strict sense. However, our present study, which used the variable sliding-window haplotype analysis (Table 4), of a large cohort has clear and strong implications. Third is the possibility that some individuals in the control 2 group (general population control) might have or develop high myopia. This may decrease the statistical power to some extent. However, since the prevalence of high myopia in Asians is reported to be 1% to 10% [35,36], the loss of the statistical power must be limited. Finally, the geographical difference of control 2 may induce potential sampling biases. However, because the Japanese population has been reported to have a rather small genetic diversity, according to data from the SNP discovery project in Japan [37], the influence of the geographical difference would be small.

In summary, we showed that none of the major tagging SNPs of *IGF-1* were associated with high and extreme myopia in a large cohort of Japanese subjects. These findings do not support the positive results of previous studies that evaluated various ethnicities. More work is required to determine the involvement of the *IGF-1* gene.
